# From short-form video addiction to burnout: exploring the role of emotional enhancement (TikTok brain) and perceived video-attributed attentional decline through the I-PACE model

**DOI:** 10.3389/fpsyg.2026.1910124

**Published:** 2026-08-20

**Authors:** Yanning Chen, Jimin Hu, Li Cheng, Muhammad Iqbal Arrasyid

**Affiliations:** 1 School of Languages and Cultures, Hunan Institute of Technology, Hengyang, Hunan, China; 2 Department of Foreign Languages, Ganzhou Teachers College, Ganzhou, Jiangxi, China; 3 Department of Management and Technology, Faculty of Management, Universiti Teknologi Malaysia, Skudai, Malaysia

**Keywords:** emotional enhancement, English-learning burnout, I-PACE model, perceived video-attributed attentional decline, short-form video addiction

## Abstract

**Inroduction:**

The rapid expansion of short-form video apps like TikTok has raised concerns about how excessive use may be associated with students’ cognitive functions, emotional control, and academic experiences. While previous research has documented associations between short-form video addiction, attentional difficulties, and adverse learning-related outcomes, there is still little research done on what specific mechanisms might explain the learning burnout of English as a Foreign Language (EFL) learners. To fill this gap, the current study examines the relationship between short-form video addiction and academic burnout of EFL learners.

**Methods:**

The theoretical framework of the I-PACE model was integrated to carry out a cross-sectional survey using validated scales with 500 Chinese undergraduates. This design tested the relationships among short-form video addiction, emotional enhancement (TikTok brain), perceived video-attributed attentional decline and English-learning burnout.

**Results:**

The quantitative analysis results using SPSS and PLS-SEM suggest that short-form video addiction is positively related to TikTok brain, perceived video-attributed attentional decline, and burnout. Furthermore, TikTok brain and perceived video-attributed attentional decline mediated the association between short-form video addiction and English-learning burnout significantly.

**Discussion:**

These empirical findings suggest that the relationship between short-form video addiction and EFL burnout may operate through both affective and cognitive pathways, providing insights into the relevance of the I-PACE model in the field of foreign language learning.

## 1 Introduction

Smartphone technology has impacted the way people live today in a fundamental manner. By the end of 2025, China’s mobile internet user base had reached a total of 1.125 billion, as per the empirical indicators (China Internet Network Information Center, 2026). This ubiquitous connectivity has triggered an explosive surge in the number of short-form video applications and consequently, the risk of behavioral addictions rapidly rises (Lu et al., 2022). Short-form video addiction can be described as the problematic use of short-form videos, which is commonly expressed as escapism, brain fatigue, and reduced functionality (Zhang et al., 2023; Yan et al., 2024). These addictive patterns often end up compromising daily life and academic achievement and intensifying psychological issues, including anxiety, a sense of failure, and academic burnout (Qu et al., 2024; Galanis et al., 2024; Haltigan et al., 2023).

Such a behavioral dependence is very pedagogically and psychologically problematic for the learners of English as a Foreign Language (EFL). From a mechanical perspective, the enhanced emotional experience brought about by watching short-form videos is known as the “TikTok brain.” It reprograms the neural mechanism of reward by stimulating the secretion of dopamine, thereby reducing students’ engagement with traditional teaching methods (Su et al., 2021; Li et al., 2025). At the same time, the overload of incoming information and changes in the structure of the platform tend to overburden limited cognitive capacity. According to the cognitive load paradigm presented by Wang and Scherr (2022), this resource depletion results in two cognitive deficiency symptoms: working memory deficiency and attentional governance deficiency, which in turn will increase the distractibility of learners in both formal learning and self-study. Finally, this cognitive-affective depletion directly connects with the main components of scholastic burnout, such as emotional exhaustion, reduced learning volition, and decreased self-efficacy (Yu et al., 2022).

Previous studies have indicated that EFL learners commonly experience challenges related to sustained attention, cognitive effort, and emotional regulation during language learning. For example, foreign language learning requires continuous practice and self-regulation, while insufficient motivation and maladaptive emotional experiences may contribute to learning burnout (Pekrun, 2006; Yu et al., 2022); there are still some notable research gaps. First, the existing literature is largely descriptive-correlational, the exact mechanisms underlying academic burnout are not systematically explained, such as how they are related to perceived video-attributed attentional decline or the TikTok brain. Previous studies have mainly focused on the general psychological consequences of overuse of short-form videos (Gong and Tao, 2024; Ye et al., 2023; Zhang et al., 2023); the relationship between excessive short-form video watching and the specific characteristics of second-language learning, such as the need for sustained cognitive endurance and strong emotional control, has not been sufficiently investigated. Third, the TikTok brain is a significant result of using addictive short-form video platforms, yet it has surprisingly not been considered as an important predictor in predictive models of academic burnout.

Therefore, the research objectives of this study are as follows:

(1) Examine the direct relationships among short-form video addiction, TikTok brain, perceived video-attributed attentional decline, and burnout among EFL learners.
(2) Investigate the mediating roles of TikTok brain and perceived video-attributed attentional decline between short-form video addiction and burnout.

## 2 Literature review and hypotheses

### 2.1 The I-PACE model

**Figure 1:**
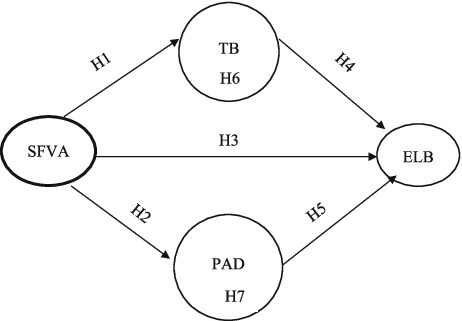
The I-PACE framework for SFVA.

I-PACE model originally conceptualized by Brand et al. (2016), offers a solid theory for understanding the development and maintenance of internet-use disorders. According to this paradigm, these disorders are developing from the interaction between predisposing individual traits, affective reactivity, cognitive biases and executive control. Importantly, the model does not assume that spending time in hyper stimulatory digital environments automatically results in negative impacts but suggests that such excessive exposure can relate to a gradual reconfiguration of neural processes in emotional and cognitive regulation, leading to dysfunctions in learning, work, and social–emotional functioning (Brand et al., 2019). Based on this theoretical framework, the present study systematically draws the relationship between short-form video addiction, TikTok brain, and perceived video-attributed attentional decline, which may eventually associate with academic burnout as conceptualized in Figure 1, in the specialized context of EFL context.

### 2.2 Hypotheses

Short-form videos are specifically designed for affective engagement. These system characteristics continuously activate the neurobiological reward system of the user, and thus promote a psychological tendency for instant gratification. Short-form video addiction (SFVA) refers to a maladaptive pattern of excessive and uncontrollable engagement with short-form video platforms (Ye et al., 2022). According to the previous literature, addictive social networking behaviors are intimately related to dopaminergic reinforcement processes and increased hedonic sensations (Tian et al., 2023). Driven by a persistent quest for validation and emotional reassurance through short-form video consumption, individuals exhibit an increased propensity for prolonged usage to prolong their heightened emotional valence (Yang, 2023). According to I-PACE, the exposure to the reward-based media environment can shape affective dependency, which may eventually develop one’s TikTok brain (TB), a psychological state with enhanced emotions, increased reward sensitivity, and increased need for high stimulation inputs (Nong et al., 2023). Therefore, the following hypothesis is put forward for this study:

*H1*: Short-form video addiction is correlated to emotional enhancement (TikTok brain) positively.

The I-PACE model indicates that problematic internet usage is systematically associated with deficiencies in executive control capabilities, specifically manifested as insufficient attention regulation and inhibition abilities (Brand et al., 2016). In short-form video contexts, users are constantly exposed to all kinds of fragmentary information, quickly changing attention and a high number of novel stimuli, which all encourage superficial heuristics instead of prolonged, deep cognitive processing (Siy et al., 2026). People who watch short-form videos for a long time are accustomed to the back-and-forth switching of information, which may impair their attention in formal education or non-digital environments. Increasing empirical studies have identified a link between excessive online media use and attention deficits. Ophir et al. (2009) showed that people who are chronically exposed to digital media presented at a rapid pace and over multiple channels have less attentional control and the ability to ignore irrelevant information is deficient. In a similar way, Liao (2024) stated that addictive media usage related to poor attentional functioning, as users become psychologically dependent on frequent emotional stimuli and have come to value a constant way of viewing social media. Additionally, Brand et al. (2024) confirmed that after extended media exposure, there are concentration deficits due to habituated distractibility. This empirical pattern is also applicable to the field of short-form video research; indeed, Chen et al. (2026) found that addictive behaviors relating to short-form videos are strongly linked to a broad range of self-regulation, attention, and general executive functioning deficits. So addictive short-form video consumption is hypothesized to serve as a major factor predicting the decline in attention:

*H2*: There is a positive relationship between short-form video addiction and perceived video-attributed attentional decline.

These emotional and cognitive vulnerabilities have deep consequences on the educational environment. The I-PACE suggests that when cognitive control abilities decline, this emotional dependence is bound to influence functional performance in real life, including an individual’s academic performance and mental health (Brand et al., 2019). Kuss and Griffiths (2017) highlighted that the more aggressively social media is used, the more psychologically fragile one becomes, the lower the academic engagement is, and the more obvious the symptoms of burnout are. English-learning burnout (ELB) refers to a state of emotional exhaustion, reduced engagement, and negative attitudes experienced by learners as a consequence of prolonged academic demands and insufficient psychological resources during English-learning activities (Huang, 2025).

Previous study verified that being in an overly stimulating, instant gratification media world erodes the tolerance of a learner for low-stimulation academic pursuits (Mao and Liao, 2025). The hedonic value of short-form videos makes students easily fall into boredom, demotivation, and emotional exhaustion in the face of high-cognitive-demand English learning activities, thereby generating resistance.

Furthermore, second language acquisition essentially requires long-term investment of cognitive resources, in-depth information processing, and continuous concentration. When executive control fails in attention management, learning efficiency declines, contributing to psychological fatigue, manifested as frustration, cognitive overload, and emotional exhaustion. Empirical research shows that attention deficits, academic fatigue, and academic burnout are closely related (Kirschner and De Bruyckere, 2017; Maslach et al., 2001).

Given these considerations, we hypothesize that:

*H3*: Short-form video addiction is positively associated with burnout in learning English.

*H4*: TikTok brain is positively associated with burnout in learning English.

*H5*: Perceived video-attributed attentional decline is positively related to burnout in learning English.

Aligned with I-PACE model, problematic digital behaviors engender emotional dependence while concurrently disrupting cognitive control mechanisms, may ultimately impair real-world functional performance (Servidio, 2021). Therefore, to indirectly test academic burnout, there is a parallel affective and cognitive path that can be taken through short-form video addiction.

Specifically, the I-PACE suggests that repeated exposure to media content that triggers the reward system can create an emotional dependency and change affective processing tendencies (Luo et al., 2022). Those who have accustomed themselves to the high emotion in daily life have significantly less psychological stamina when performing effort and reflection learning tasks (Lin et al., 2026). This empirical mechanism is supported by the literature. Montag et al. (2021) demonstrated that the design of TikTok, which promoted the formation of affective engagement and reward-seeking cycles, reshaped the cognitive-affective processing frameworks. Moreover, Sun and Zhang (2021) showed that excessive short-form video consumption led to maladaptive preferences for immediate gratification, seriously affecting persistence in tasks that required long cognitive processing. Contextualized within second language acquisition (SLA), mastering English demands sustained focus and protracted psychological investment. Learners habituated to low-level affective gratification are highly susceptible to premature boredom and fatigue, which systematically exacerbates manifestations of burnout (Pekrun, 2006). Consequently, TikTok brain may function as an effective mediator.

Secondly, perceived video-attributed attentional decline is conceptualized as a parallel cognitive mediator. Within the framework of the I-PACE, Sun (2023) suggested a systematic relationship between addictive online behaviors and decrements in attention and executive functioning. Sloppy and fragmented information, as well as the quick turnover of material, creates long-term problems with sustained attention for users of the short video format. This is in line with the broad literature on the influences of digital media, with Ophir et al. (2009) finding a strong correlation between the frequent use of fast-changing media and reduced attentional control, and Kuss and Griffiths (2017)’s finding that addictive social media users often experience deficits in concentration. Moreover, Firth et al. (2019) stated that overuse of digital media contributes to an increased level of cognitive overload and chronic distraction. Since learning English requires long-term concentration, various modalities of attentional deficits generate a profound sense of exhaustion, subsequently culminating in scholastic burnout (Mao and Liao, 2025). Accordingly, it is hypothesized that attentional decline serves as a pivotal cognitive trajectory.

*H6*: TikTok brain mediates the relationship between short-form video addiction and English-learning burnout.

*H7*: Perceived video-attributed attentional decline mediates the relationship between short-form video addiction and English-learning burnout.

## 3 Methodology

### 3.1 Procedure

This study adopts a combination of purposive sampling and snowball sampling, with a focus on recruiting people who have long used short videos for participation in the survey. To ensure the validity and relevance of the research sample, clear entry criteria were set for the survey: all respondents must have studied English within the Chinese educational context and have completed at least one semester or more of English course training, with a stable English learning experience. Participants who did not meet these criteria were screened out at the beginning. The questionnaire was administered in English because all measurement scales were originally developed and validated in English, and the participants were EFL learners with prior English-learning experience in the Chinese educational context. To ensure the clarity and comprehensibility of the items, bilingual researchers independently reviewed the items and examined their Chinese interpretations before data collection. Any potential ambiguities in wording or meaning were discussed and resolved. Therefore, the original English versions of the scales were retained to maintain conceptual consistency and avoid potential deviations caused by translation.

Before the formal survey, a two-phase pilot study was conducted to further verify the clarity and applicability of the questionnaire. First, two professors specializing in educational psychology and English language education reviewed the questionnaire to evaluate its content validity and identify potential issues in item expression. Subsequently, a pre-test was conducted with 30 EFL learners recruited through online communities (e.g., QQ Groups) to examine whether participants could accurately understand the English items and to identify any unclear wording. The feedback from the pilot study indicated that the participants were able to comprehend the questionnaire without the need for a Chinese version, and minor revisions were made accordingly. The final questionnaire was then administered through the Wenjuanxing platform. Participants were informed that the survey required approximately 10–15 min to complete, and a small incentive (5 RMB) was provided upon submission of a valid response. The survey included multiple constructs related to short-form video addiction, pleasure, concentration, and learning outcomes. Although parts of this dataset have been used in previous studies, the present study examines distinct research objectives by focusing on the relationship between short-form video addiction and English-learning burnout from the perspective of the I-PACE model.

Descriptive, internal consistency, and bivariate correlation analyses were performed using SPSS 27 to evaluate the primary latent constructs. Hypothesis testing and structural model estimation was performed using Partial Least Squares Structural Equation Modeling (PLS-SEM) with SmartPLS 3.3.9 PLS-SEM was considered to be the most suitable statistical tool since the main analytical goal is to investigate and predict the structural model with the magnitude and direction of the path coefficients of the hypothesized structural network and not to strictly confirm an already known model (Hu et al., 2026). This approach can be very effective in theory building, model development and the simultaneous estimation of complex mediating pathways. In contrast, Covariance-Based Structural Equation Modeling (CB-SEM) is generally used only for confirmatory analysis and goodness-of-fit and is very sensitive to the assumption of multivariate normality (Kline, 2023).

Based on *a priori* statistical power analysis performed using G*Power 3.1 with parameters of statistical power level = 95% and a significance alpha (*p*-value) = 0.05, the number of respondents needed for this study was ≥ 119 participants in a five-pathway, three predictors in the complex endogenous constructs’ structural configuration with an effect size (*f*^2^) of 0.15 (Cohen, 1988). A total of 559 questionnaires were initially collected. During the data-cleaning process, respondents who did not meet the criteria regarding English-learning experience, failed the screening question, exhibited careless responses, or completed the questionnaire in a short time were excluded. After removing these invalid responses, 500 valid questionnaires were retained for the final analysis. This sample size significantly exceeds the G*Power recommended sample size and the 200-response benchmark that is suggested for stability of PLS-SEM estimations (Hair et al., 2021).

Demographic profiling of the finalized dataset showed a good balance of males and females, with 51.4% of females and 48.6% males. The distribution of the academic standing of the cohort was senior (24%), junior (22.8%), sophomore (24.6%), and freshman (28.6%). In terms of how often people use short video applications, 18% used them 1–3 times a week, 29.6% used them 4–6 times a week, and over half of the respondents (52.4%) used them every day. Also, the difference in metrics for daily consumption time was broken down into <2 h (24.2%), 2–3 h (31.6%), 3–4 h (21.2%), and >4 h (23%).

### 3.2 Measurement

Short-form video addiction (SFVA) was assessed by the 10-item instrument developed by Ye et al. (2022), while the emotional enhancement (TikTok brain) was measured on the basis of a 9-item scale created by Ye et al. (2025). At the same time, a 6-item framework by Ye et al. (2025) was used to measure perceived video-attributed attentional decline (PAD), while a 10-item English-learning burnout inventory was developed by Huang (2025) to measure ELB. The scales developed by Ye et al. (2025) were selected because they represent the first empirically validated instruments specifically designed to measure TikTok brain and perceived video-attributed attentional decline in the context of short-video use. Although the TikTok brain scale refers specifically to TikTok short videos, TikTok was treated as a representative short-form video environment in the present study. In contrast, SFVA and PAD were measured using the broader term “short videos” because these constructs aim to capture general patterns of short-form video consumption and related attentional experiences across platforms. All scales (see Appendix Table A) were adapted through minor wording revisions to fit the English-learning context (e.g., replacing general learning references with English-learning situations) while retaining the original construct definitions and item structure. No items were added or deleted. Responses from the participants were indexed using 5-point Likert Scale ranging from 1 (strongly disagree) to 5 (strongly agree).

## 4 Results

### 4.1 Measurement model

Cronbach’s alpha is the most well-known and widely used indicator for assessing reliability. In this study, the Cronbach’s alpha of each construct exceeded 0.7, indicating good reliability. However, some scholars believe that the Cronbach’s alpha underestimates the internal consistency of the constructs (Chin, 1998). Therefore, Geldhof et al. (2014) proposed to adopt composite reliability as an alternative strategy to make up for the shortcomings of the Cronbach’s alpha. The composite reliability (CR) of this study was all above 0.7. Thus, all constructs have a high level of reliability (Hair et al., 2019). In addition, the convergent validity was well established, with all standardized item factor loadings above 0.70 (the minimum value), and all average variance extracted (AVE) values well above 0.50 (the benchmark) (Hair et al., 2019) with values tabulated in full in Table 1.

**Table 1:** Measurement model statistics.

Construct	Factor loading	Cronbach’s alpha	Composite reliability	Average variance extracted
SFVA	0.715–0.823	0.895	0.917	0.585
TB	0.792–0.823	0.933	0.944	0.650
PAD	0.779–0.824	0.892	0.917	0.648
ELB	0.707–0.763	0.901	0.918	0.529

Discriminant validity is used to verify whether there is a statistically significant difference in the correlation between two different constructs. The Fornell-Larcker criterion is a commonly used method for assessing discriminant validity (Fornell and Larcker, 1981). However, Henseler et al. (2015) demonstrated through simulation studies that in common research scenarios, the Fornell-Larcker criterion cannot reliably detect the absence of discriminant validity. Therefore, they proposed an alternative method based on a multi-attribute-multi-method matrix to assess discriminant validity, namely the heterogeneity ratio of correlation coefficients for three-attribute-single-attribute (HTMT). Systematically, both the Fornell-Larcker criterion and the HTMT ratio of correlations (as reported in Table 2) confirmed the discriminant validity. In fact, the square root of the AVE of each latent variable was greater than the maximum bivariate correlation between it and the other latent variable. At the same time, all the HTMT ratios were kept well below the conservative limit of 0.90 (Henseler et al., 2015; Henseler et al., 2016), supporting the empirical separateness and statistical independence of the psychometric scales. Finally, the comprehensive indicator for measuring the degree of data fitting is the standardized root mean square residual (SRMR), which represents the standardized difference between the true correlation and the estimated correlation (Hu and Bentler, 1998). In this study, SRMR was examined as a measure of model fit in the context of a cross-sectional design. Consequently, the resulting estimation was within the well-established critical value of 0.10 (Hu and Bentler, 1999; Hair and Alamer, 2022), indicating good fit between the proposed structural model and the data (Table 3).

**Table 2:** Discriminant validity.

Fornell-Larcker criterion					HTMT			
Construct	1	2	3	4	1	2	3	4
1. PAD	0.805							
2. ELB	0.265	0.727			0.287			
3. SFVA	0.300	0.369	0.765		0.329	0.389		
4. TB	0.212	0.206	0.196	0.807	0.226	0.219	0.212	

**Table 3:** Model fit.

Fit indices	Saturated model	Estimated model
SRMR	0.061	0.068
Chi-Square	2099.355	2109.677
NFI	0.801	0.800

### 4.2 Structural model

**Figure 2:**
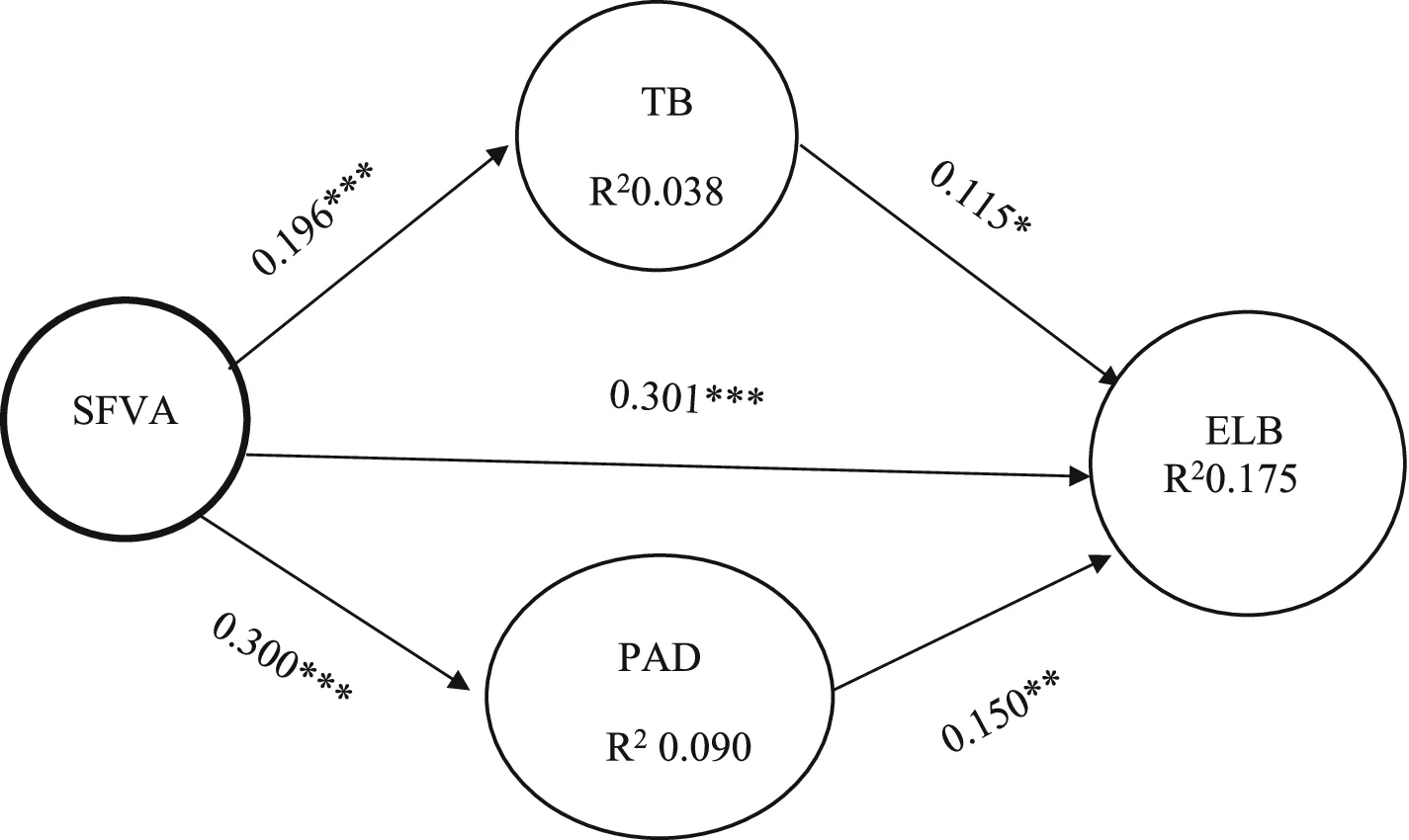
Structural model. ***p < 0.001, **p < 0.01, *p < 0.05.

Before examining the structural relationships, this study employed the Kock (2015) fully collinearity VIF method for assessment. All values were below 3.3, thus eliminating the common method bias. The VIF values of both the inner and outer values were below the threshold, indicating no multiple collinearity issues (see Appendix Tables B, C, respectively). The structural model is used to evaluate the associations between the latent variables hypothesized after confirming the goodness of fit of the measurement model. It assesses the significance of the proposed direct and indirect paths within the theoretical framework (Hair and Alamer, 2022). Figure 2 shows the results of the structural model executed using a bootstrapping procedure with 5,000 resamples to evaluate the proposed hypotheses. As detailed in Table 4, SFVA positively related to TB (*β* = 0.196, *p* < 0.001), PAD (*β* = 0.300, *p* < 0.001), and ELB (*β* = 0.301, *p* < 0.001), thereby validating H1, H2, and H3. Although the relationship between TB and ELB (*β* = 0.115, *p* < 0.05) was statistically significant supporting H4, its explanatory contribution was limited (*f*^2^ = 0.016). Lastly, PAD positively related to ELB (*β* = 0.150, *p* < 0.01), providing empirical support for H5. And the significance is determined at the 95% confidence level. The *R*^2^ was examined to assess the explanatory power of the structural model. In addition, all endogenous constructs exhibited *Q*^2^ values greater than zero (PAD: *Q*^2^ = 0.058; TB: *Q*^2^ = 0.030; ELB: *Q*^2^ = 0.077), indicating that the model retains predictive capability for these constructs (Hair et al., 2021).

**Table 4:** Structural model results.

Relationships	Beta	Sample mean	Standard deviation	*t*-value	*p*-value	*f* ^2^
H1 SFVA - > TB	0.196***	0.202	0.045	4.338	0.000	0.052
H2 SFVA - > PAD	0.300***	0.302	0.047	6.370	0.000	0.103
H3 SFVA - > ELB	0.301***	0.303	0.049	6.133	0.000	0.072
H4 TB - > ELB	0.115*	0.117	0.049	2.353	0.019	0.016
H5 PAD - > ELB	0.150**	0.153	0.056	2.676	0.008	0.027

***p < 0.001, **p < 0.01, *p < 0.05; *f*^2^ value (0.35 large effect size, 0.15 medium effect size, 0.02 small effect size, < 0.02 negligible or trivial effect size).

### 4.3 Mediation analysis

To accurately delineate the empirical sampling distribution of the indirect relationships, the hypothesized mediating pathways among the latent constructs were systematically evaluated in accordance with the bootstrapping framework formalized by Preacher and Hayes (2008). Indirect relationships were deemed statistically significant when the critical t-value was greater than 1.96 at the 5% significance level and when there was no zero between the lower limit (LL) and upper limit (UL) of the 95% bias-corrected confidence interval. The empirical results of the path estimation in Table 5 show that TB acts as the mediating role between SFVA and ELB. Likewise, the indirect pathway between these two main constructs via PAD was statistically significant, as well, and thus provides empirical support for Hypotheses 6 and 7. The total indirect relationship of SVA on ELB through TB and PAD was significant (*β* = 0.068, *p* < 0.001) accounted for 20.6% of the total effect, indicating partial mediation.

**Table 5:** Mediation analysis.

Path	Beta	Sample mean	Standard deviation	*t*-value	*p*-value	LLCI	ULCI	Result
H6 SFVA- > TB- > ELB	0.023	0.023	0.011	2.103	0.036	0.004	0.046	Supported
H7 SFVA- > PAD- > ELB	0.045	0.046	0.019	2.360	0.018	0.012	0.087	Supported
Total indirect effect	0.068	0.070	0.021	3.235	0.001	0.032	0.113	Partial mediation
VAF	20.6%							Partial mediation

### 4.4 Sensitivity analysis

To address the potential concern that SFVA items related to academic consequences may overlap conceptually with English-learning burnout, a sensitivity analysis was conducted by removing SFVA items 5 and 6, which refer to missed classes and declined academic performance. The structural model was re-estimated using the remaining SFVA indicators. The results showed that the relationship between SFVA and ELB remained significant (*β* = 0.297***, *p* < 0.001), with the coefficient decreased slightly compared with the original model (*β* = 0.301***, *p* < 0.001). These findings suggest that the association between SFVA and ELB is unlikely to be driven solely by overlapping item content. Detailed results of the sensitivity analysis are provided in Appendix Table D. Therefore, the findings indicate that the relationship between SFVA and ELB remains stable even when outcome-related indicators are excluded from the SFVA measurement.

## 5 Discussion

### 5.1 The direct relationships

Empirical results offer initial evidence supporting the application of the I-PACE model for understanding the relationships among SFVA, TB, PAD, and ELB. This study confirms that SFVA is positively associated with TB, PAD, and ELB.

Specifically, the significant positive path from SFVA to TB (H1) indicates that problematic short-form video use is related to changes in users’ emotional processing tendencies. Drawing on the I-PACE framework, the “Affect” component reflects the emotional states triggered by Internet-related stimuli (Brand et al., 2016; Brand et al., 2019). In the context of short-video consumption, users who repeatedly seek rewarding experiences may become increasingly sensitive to immediate emotional gratification. Short-form video platforms, through continuous stimulation, algorithmic recommendations, and instant feedback, create a digital environment brimming with novelty and emotional arousal (Yang, 2023). Consistent with previous research, frequent short-form video use has been associated with heightened reward sensitivity and greater reliance on digitally mediated emotional experiences (Huang et al., 2022; Chen et al., 2026). Therefore, the relationship between SFVA and TB highlights the relevance of the affective processes proposed in the I-PACE framework for understanding the psychological experiences associated with problematic short-form video use in educational contexts.

Second, the significant positive relationship between SFVA and PAD (H2) further supports the “Cognition” and “Execution” proposed in the I-PACE model. Specifically, frequent exposure to rapidly changing digital content is likely to alter an individual’s cognitive processing methods and weaken their ability to maintain attention. According to the cognitive and executive dimensions in the I-PACE model, the use of problematic internet typically manifests as a bias in cognitive processing and a decline in the level of executive control for goal-oriented behaviors (Brand et al., 2016). Existing research also supports this view; for instance, Firth et al. (2019) and Ophir et al. (2009) verified that individuals who frequently watch highly stimulating digital media often have difficulty concentrating for a long period. Studies by Ye et al. (2025) and Liu et al. (2023) also found that excessive use of short videos is significantly associated with attention problems and impaired cognitive control. Accordingly, the current results imply that SFVA could correlate with poorer attention in EFL learners, arising from cognitive overload and impaired capacity to regulate attention.

Third, the study found a positive association between SFVA and ELB (H3), demonstrating that problematic short-form video use may be related to emotional exhaustion, negative learning experiences, and cynicism during the learning process. From the perspective of the I-PACE framework, addictive behaviors may be accompanied by various psychological processes and functional impairments beyond the addictive behavior itself (Brand et al., 2016; Brand et al., 2019). Compared with many other academic tasks, English learning requires sustained attention, continuous practice, and long-term motivation. However, learners who frequently engage with highly stimulating digital content may experience greater difficulty maintaining engagement in effortful learning activities. This finding is consistent with previous studies linking problematic digital media use with academic exhaustion, reduced learning engagement, and burnout symptoms (Huang, 2025; Mao and Liao, 2025). Therefore, the present findings highlight SFVA as a meaningful correlate of English-learning burnout and provide further insights into the educational consequences associated with problematic short-form video use.

Lastly, the significant structural relationships of PAD and TB with ELB, respectively, provide empirical support for the cognitive and affective pathways proposed by the I-PACE model. English learning consumes continuous cognitive resources to sustain listening, speaking, reading, and writing practices. Students who have difficulty concentrating need to expend additional mental energy to counteract distractions, which may be associated with greater mental fatigue and lower learning efficiency. This explanation coincides with existing evidence linking perceived attentional decline to cognitive depletion and learning burnout (Huang, 2025; Koudsia and Kirchner, 2024). Meanwhile, as emphasized by the I-PACE model, emotional reactions associated with compulsive digital use can guide subsequent behaviors and psychological states (Brand et al., 2019). Learners reliant on instant emotional gratification often struggle to stay motivated amid tough learning assignments. In line with Pekrun (2006), students are prone to feel bored and drained when academic tasks fail to provide comparable levels of emotional engagement, which are all the characteristics of English-learning burnout.

### 5.2 The mediate relationships

Along with the above direct relationships, this study also confirmed that TB and PAD partially mediate the relationship between SFVA and ELB. The above research results provide empirical evidence for the process-based assumptions of the I-PACE model. This model posits that problematic internet behaviors do not merely stem from direct behavioral relationships alone but are the results among affective responses, cognitive processes, and executive-control functions (Brand et al., 2019). In particular, the TB more often manifests as the affective pathway that links addiction with learning burnout. According to the “Affect” component of the I-PACE model, repeatedly watching can strengthen emotional responses toward specific online activities. As previous studies demonstrated that the algorithm structure of TikTok platform continuously reinforces the behavior of seeking rewards (Ye et al., 2025; Montag et al., 2021). In educational contexts, this emotional dependence may lower the learners’ tolerance for delayed rewards and sustained effort, making English-learning activities seem less attractive. Therefore, TB can provide the potential explanation for how SFVA may be associated with learning burnout, breaking through the limitation of merely analyzing viewing duration.

PAD’s mediating role further highlights the relevance of cognitive and executive-control processes within the I-PACE model. In line with the “Cognition” and “Execution” dimensions, overconsumption of short videos can split people’s attention and weaken their capacity to manage goal-driven behaviors. Existing studies have confirmed that individuals who excessively use short-form videos usually have attentional problems and difficulty in sustained learning (Ye et al., 2025; Liu et al., 2023). When learners have difficulty maintaining their concentration, they may need additional cognitive resources to resist distractions and complete their academic tasks. This additional cognitive burden can generate burnout and may ultimately relate to poor academic performance (Adrian et al., 2016; Rebetez et al., 2018). Thus, PAD represents a potential cognitive pathway linking SFVA to ELB.

### 5.3 Theoretical and practical implications

#### 5.3.1 Theoretical implications

This study has several theoretical contributions by applying the I-PACE model to short-form video addiction and English-learning burnout among EFL learners. While the I-PACE model is often used to explain various internet addiction behaviors such as social media and smartphone use (Brand et al., 2019), its value in digital education contexts remains relatively underexplored. This study integrates the affective and cognitive mechanisms corresponding to SFVA, suggesting that the I-PACE framework can explain how digital media overuse is associated with learning burnout.

Secondly, this study examines the TB as a potential emotion-enhancing mechanism, thereby explaining the link between SFVA and learning burnout, enriching the “affect” component of the I-PACE model. Previous research generally suggests that the TB may reflect an individual’s high preference for immediate rewards and rapid emotional stimulation (Montag et al., 2021; Ye et al., 2025), but the role of this variable in the field of education requires further empirical support. This study operationalizes the TB as emotional enhancement, providing empirical evidence for its association with English-learning burnout, suggesting that the emotional feedback people experience in stimulating digital environments is an important psychological process within the I-PACE system. Therefore, the association between SFVA and learning burnout may involve not only excessive usage patterns but also individual differences in emotional responses and reward-related tendencies.

Third, this study also explains the cognitive and executive dimensions of the I-PACE model, confirming that PAD can act as a cognitive mediator, establishing a pathway between SFVA and ELB. This conclusion aligns with previous research that excessive exposure to digital media impairs an individual’s ability to regulate attention (Ophir et al., 2009; Firth et al., 2019). Besides, this study investigates two potential mediating pathways through TikTok brain and perceived video-attributed attentional decline, providing further insights into the application of the I-PACE model in explaining EFL learners’ burnout.

#### 5.3.2 Practical implications for higher education

The findings of this study provide several practical implications for higher education institutions. First, universities need to move beyond simply regulating students’ screen hours and place more emphasis on the emotional and cognitive consequences of problematic short-form video usage. To put this into practice, universities can integrate training on digital literacy and digital wellness into student development curricula to help students understand how excessive exposure to highly stimulating digital environments may influence attention regulation, emotional states, and learning motivation. Second, workshops and training sessions centered on self-directed media management and sustainable digital routines can also guide students to build healthier interactions with online video platforms. Furthermore, the campus mental health center can regard the excessive use of short videos as a warning sign of students’ learning difficulties, academic pressure, etc. They can incorporate the assessment of digital media behavior into their regular support systems and implement personalized intervention measures to help students enhance their self-discipline, attention control abilities, and emotional management skills.

#### 5.3.3 Practical implications for EFL learning

These findings are also of great significance for the teaching and learning English as a foreign language. English teachers should be aware that students’ lack of enthusiasm or feeling tired during the learning process may partly stem from their digital media usage habits. Integrating digital self-management skills into language teaching, such as encouraging students to record their short-video usage and cultivating better time management habits, can help them stay focused and better maintain their attention when learning English. Besides, EFL teachers may consider designing learning activities that enhance students’ engagement and intrinsic motivation to reduce their reliance on immediate digital gratification. For example, meaningful task design, achievable learning goals, and interactive learning experiences may increase students’ sense of competence and involvement in language learning. By promoting healthier digital behaviors and strengthening learners’ capacity for sustained attention, educators may better support students in adapting to the demands of English learning in the digital era and addressing learning burnout-related difficulties.

### 5.4 Limitations and future research directions

First, the purposive sampling combined with snowball sampling might influence the generalizability of the research findings. Although the methods enabled us to obtain a considerable amount of valid data, they might be prone to self-selection bias, and sample’s representativeness may be compromised. The participants in this study were all EFL learners from Chinese universities. Therefore, when applying the conclusions to other populations, caution is necessary. In future research, it is recommended to adopt probability sampling techniques and try to recruit more participants from different educational backgrounds, age groups, and cultural environments to improve the research results’ external validity.

Second, this study does not include certain control variables that might relate to English-learning burnout. Although our model demonstrates reasonable explanatory power, future studies should incorporate relevant demographic, academic, and psychological control variables to more rigorously assess the extent to which short-form video addiction influences learning burnout. This approach also helps eliminate potential confounding factors, making research findings more reliable and robust.

Third, the cross-sectional design limits the ability to determine the temporal sequence and causal relationships among SFVA, TB, PAD, and ELB. Although the structural equation model supports the proposed associations and mediation paths, the underlying developmental processes require further validation.

Fourth, the measurement of PAD may involve attribution bias because the items refer to changes in attention “since using short video apps,” potentially implying a predetermined link between short-form video use and attention decline. Therefore, these findings should be interpreted cautiously. Future studies should employ longitudinal designs, experimental interventions, and objective measures (e.g., behavioral tasks or neurocognitive assessments) to further examine these relationships.

Finally, the measurement of the TB is still in its early stages of development. Although this study defines the TB as enhanced emotional response, this concept is based on self-reported cognition. This current measurement may be more reflective of subjective experiences than objective neurocognitive changes, according to previous findings (Ye et al., 2025). Future research could combine self-report scales with behavioral, physiological, or neurocognitive indicators (such as attention tasks, eye-tracking measurements, or neuroimaging techniques) to improve the validity of TB assessments. Furthermore, multidimensional measurement methods may help reveal the underlying structure of this emerging construct.

## 6 Conclusion

From SFVA to burnout, the results supported two mediators of TB and PAD in explaining foreign language learners’ burnout based on I-PACE model. To be more specific, the findings indicate that SFVA is not only linked with TB positively, but also related to PAD and ELB. Besides, TB was significantly associated with ELB, although the effect size was relatively small. And PAD is also an explanatory factor of burnout. In addition, TB and PAD both partially explained the association between SFVA and burnout, suggesting that the association between addictive short-form video use and EFL burnout may operate through affective and cognitive pathways. These findings give insights into the application scope of the I-PACE model to foreign language learning and provides empirical evidence for the new concept of TikTok brain. From a practical application perspective, our results remind educators and universities to devote more care to students’ daily digital media usage habits and to formulate and execute interventions to help students use media more healthily, better regulate their attention, and thereby improve their overall learning state.

## Data Availability

The original contributions presented in the study are included in the article/Supplementary material, further inquiries can be directed to the corresponding author.

## References

[ref1] Adrian M. Leonard R. Christine E. M. (2016). "FacELBocrastination"? Predictors of using Facebook for procrastination and its effects on students' well-being. Comput. Hum. Behav. 64, 65–76. doi: 10.1016/j.chb.2016.06.011

[ref2] Brand C. Fochesatto C. F. Gaya A. R. Schuch F. B. López-Gil J. F. (2024). Scrolling through adolescence: unveiling the relationship of the use of social networks and its addictive behavior with psychosocial health. Child Adolesc. Psychiatry Ment. Health 18:107. doi: 10.1186/s13034-024-00805-0, 39217325 PMC11365153

[ref3] Brand M. Wegmann E. Stark R. Müller A. Wölfling K. Robbins T. W. . (2019). The interaction of person-affect-cognition-execution (I-PACE) model for addictive behaviors: update, generalization to addictive behaviors beyond internet-use disorders, and specification of the process character of addictive behaviors. Neurosci. Biobehav. Rev. 104, 1–10. doi: 10.1016/j.neubiorev.2019.06.032 31247240

[ref4] Brand M. Young K. S. Laier C. Wölfling K. Potenza M. N. (2016). Integrating psychological and neurobiological considerations regarding the development and maintenance of specific internet-use disorders: an interaction of person-affect-cognition-execution (I-PACE) model. Neurosci. Biobehav. Rev. 71, 252–266. doi: 10.1016/j.neubiorev.2016.08.033 27590829

[ref5] Chen Y. Hu J. Cheng L. (2026). Academic burnout in the TikTok era: a battle between pleasure and concentration. Front. Psychol. 17:1774030. doi: 10.3389/fpsyg.2026.1774030, 41867980 PMC12999888

[ref6] Chin W. W. (1998). Commentary: issues and opinion on structural equation modeling. MIS Q. 22, 7–16.

[ref7] China Internet Network Information Center (2026) China's Internet user base hits 1.125 Billion as AI Adoption Surges. The State Council of the People's Republic of China. Available online at: http://english.www.gov.cn/archive/statistics/202602/05/content_WS698442cac6d00ca5f9a08edc.html (Accessed February 5, 2026).

[ref8] Cohen J. (1988). Statistical Power Analysis for the Behavioral Sciences. Hillsdale: Routledge.

[ref9] Firth J. Torous J. Stubbs B. Firth J. A. Steiner G. Z. Smith L. . (2019). The "online brain": how the internet may be changing our cognition. World Psychiatry 18, 119–129. doi: 10.1002/wps.20617, 31059635 PMC6502424

[ref10] Fornell C. Larcker D. F. (1981). Evaluating structural equation models with unobservable variables and measurement error. J. Mark. Res. 18, 39–50. doi: 10.1177/002224378101800104

[ref11] Galanis P. Katsiroumpa A. Moisoglou I. Konstantakopoulou O. (2024). The TikTok addiction scale: development and validation. AIMS Public Health 11, 1172–1197. doi: 10.3934/publichealth.2024061, 39802558 PMC11717542

[ref12] Geldhof G. J. Preacher K. J. Zyphur M. J. (2014). Reliability estimation in a multilevel confirmatory factor analysis framework. Psychol. Methods 19, 72–91. doi: 10.1037/a0032138, 23646988

[ref13] Gong Q. Tao T. (2024). The relationship between short video usage and academic achievement among elementary school students: the mediating effect of attention and the moderating effect of parental short video usage. PLoS One 19:e0309899. doi: 10.1371/journal.pone.0309899, 39585867 PMC11588245

[ref14] Hair J. Alamer A. (2022). Partial least squares structural equation modeling (PLS-SEM) in second language and education research: guidelines using an applied example. Res. Methods Appl. Linguist. 1:100027. doi: 10.1016/j.rmal.2022.100027

[ref15] Hair J. F. Black W. C. Babin B. J. Anderson R. E. Tatham R. L. (2019). Multivariate Data Analysis. 8th Edn. Andover: Cengage.

[ref16] Hair J. F. Jr. Hult G. T. M. Ringle C. M. Sarstedt M. Danks N. P. Ray S. (2021). “An introduction to structural equation modeling,” in Partial Least Squares Structural Equation Modeling (PLS-SEM) Using R: a Workbook, (Cham: Springer), 1–29.

[ref17] Haltigan J. D. Pringsheim T. M. Rajkumar G. (2023). Social media as an incubator of personality and behavioral psychopathology: symptom and disorder authenticity or psychosomatic social contagion? Compr. Psychiatry 121:152362. doi: 10.1016/j.comppsych.2022.152362 36571927

[ref18] Henseler J. Hubona G. Ray P. A. (2016). Using PLS path modeling in new technology research: updated guidelines. Ind. Manag. Data Syst. 116, 2–20. doi: 10.1108/IMDS-09-2015-0382

[ref19] Henseler J. Ringle C. M. Sarstedt M. (2015). A new criterion for assessing discriminant validity in variance-based structural equation modeling. J. Acad. Mark. Sci. 43, 115–135. doi: 10.1007/s11747-014-0403-8

[ref20] Hu L. Bentler P. M. (1998). Fit indices in covariance structure modeling: sensitivity to under parameterized model misspecification. Psychol. Methods 3, 424–453. doi: 10.1037/1082-989X.3.4.424

[ref21] Hu L. Bentler P. M. (1999). Cutoff criteria for fit indexes in covariance structure analysis: conventional criteria versus new alternatives. Struct. Equ. Model. 6, 1–55. doi: 10.1080/10705519909540118

[ref22] Hu J. Chen Y. Amrollahifar J. (2026). From empathy and self-disclosure to mimicry consumption: exploring social media influencers' influence mechanisms through the heuristic-systematic model. Asia Pac. J. Mark. Logist. 38, 1–18. doi: 10.1108/APJML-08-2025-1590

[ref23] Huang K. (2025). Unveiling the complex mechanism of short video addiction on English learning engagement and burnout among college EFL students. Research Square, 1–27. doi: 10.21203/rs.3.rs-5885393/v1

[ref24] Huang Q. Hu M. Chen H. (2022). Exploring stress and problematic use of short-form video applications among middle-aged Chinese adults: the mediating roles of duration of use and flow experience. Int. J. Environ. Res. Public Health 19:132. doi: 10.3390/ijerph19010132, 35010389 PMC8751076

[ref25] Kirschner P. A. De Bruyckere P. (2017). The myths of the digital native and the multitasker. Teach. Teach. Educ. 67, 135–142. doi: 10.1016/j.tate.2017.06.001

[ref26] Kline R. B. (2023). Principles and Practice of Structural Equation Modeling. 5th Edn. New York: The Guilford Press.

[ref27] Kock N. (2015). Common method bias in PLS-SEM: a full collinearity assessment approach. Int. J. e-Collab. 11, 1–10. doi: 10.4018/IJEC.2015100101

[ref28] Koudsia S. Kirchner M. (2024). Reducing cognitive overload for students in higher education: a course design case study. J. High. Educ. Theory Pract. 24, 97–124. doi: 10.33423/jhetp.v24i10.7382

[ref29] Kuss D. J. Griffiths M. D. (2017). Social networking sites and addiction: ten lessons learned. Int. J. Environ. Res. Public Health 14:311. doi: 10.3390/ijerph14030311, 28304359 PMC5369147

[ref30] Li M. Huang H. Zhou K. Meng M. (2025). Unraveling the neural dichotomy of consensus and idiosyncratic experiences in short video viewing. Brain Cogn. 184:106260. doi: 10.1016/j.bandc.2024.106260 39756094

[ref31] Liao M. (2024). Analysis of the causes, psychological mechanisms, and coping strategies of short video addiction in China. Front. Psychol. 15:1391204. doi: 10.3389/fpsyg.2024.1391204, 39165759 PMC11333346

[ref32] Lin Y. Mo C. Wei B. (2026). From immersion to burnout: anxiety mechanisms and pathways to motivational exhaustion in gamified health education. Front. Public Health 14:1732924. doi: 10.3389/fpubh.2026.1732924, 41717600 PMC12913498

[ref33] Liu Z. Hu R. Bi X. (2023). The effects of social media addiction on reading practice: a survey of undergraduate students in China. J. Doc. 79, 670–682. doi: 10.1108/JD-05-2022-0111

[ref34] Lu L. Liu M. Ge B. Bai Z. Liu Z. (2022). Adolescent addiction to short video applications in the mobile internet era. Front. Psychol. 13:893599. doi: 10.3389/fpsyg.2022.893599, 35619797 PMC9127662

[ref35] Luo R. Li Q. Meng G. Zheng Y. Hu K. Zhang X. . (2022). The association between intolerance of uncertainty and internet addiction during the second wave of the coronavirus disease 2019 pandemic: a multiple mediation model considering depression and risk perception. PsyCh J. 11, 383–391. doi: 10.1002/pchj.545, 35385213 PMC9088591

[ref36] Mao M. Liao F. (2025). Undergraduates short form video addiction and learning burnout association involving anxiety symptoms and coping styles moderation. Sci. Rep. 15:24191. doi: 10.1038/s41598-025-09656-x, 40624123 PMC12234783

[ref37] Maslach C. Schaufeli W. B. Leiter M. P. (2001). Job burnout. Annu. Rev. Psychol. 52, 397–422. doi: 10.1146/annurev.psych.52.1.397, 11148311

[ref38] Montag C. Yang H. Elhai J. D. (2021). On the psychology of TikTok use: a first glimpse from empirical findings. Front. Public Health 9:641673. doi: 10.3389/fpubh.2021.641673, 33816425 PMC8010681

[ref39] Nong W. He Z. Ye J. H. Wu Y. F. Wu Y. T. Ye J. N. . (2023). The relationship between short video flow, addiction, serendipity, and achievement motivation among Chinese vocational school students: the post-epidemic era context. Healthcare 11:462. doi: 10.3390/healthcare11040462, 36832995 PMC9957412

[ref40] Ophir E. Nass C. Wagner A. D. (2009). Cognitive control in media multitaskers. Proc. Natl. Acad. Sci. 106, 15583–15587. doi: 10.1073/pnas.0903620106, 19706386 PMC2747164

[ref1101] Preacher K. J. Hayes A. F. (2008). Asymptotic and resampling strategies for assessing and comparing indirect effects in multiple mediator models. Behav. Res. Methods. 40, 879–891. doi: 10.3758/BRM.40.3.879, 18697684

[ref41] Pekrun R. (2006). The control-value theory of achievement emotions: assumptions, corollaries, and implications for educational research and practice. Educ. Psychol. Rev. 18, 315–341. doi: 10.1007/s10648-006-9029-9

[ref42] Qu D. Liu B. Jia L. Zhang X. Chen D. Zhang Q. . (2024). The longitudinal relationships between short video addiction and depressive symptoms: a cross-lagged panel network analysis. Comput. Hum. Behav. 152:108059. doi: 10.1016/j.chb.2023.108059

[ref43] Rebetez M. M. L. Rochat L. Barsics C. Van der Linden M. (2018). Procrastination as a self-regulation failure: the role of impulsivity and intrusive thoughts. Psychol. Rep. 121, 26–41. doi: 10.1177/0033294117720695, 28776482

[ref44] Servidio R. (2021). Fear of missing out and self-esteem as mediators of the relationship between maximization and problematic smartphone use. Curr. Psychol. 42, 1–11. doi: 10.1007/s12144-020-01341-833519148

[ref45] Siy C. Jung K. Song S. Lee K. H. Kim J. (2026) Endless Swipes and Recommendations: the Impact of Short-form Video Platforms on Context-Switching and Children's Working Memory Proceedings of the 2026 CHI Conference on Human Factors in Computing Systems

[ref46] Su C. Zhou H. Gong L. Teng B. Geng F. Hu Y. (2021). Viewing personalized video clips recommended by TikTok activates default mode network and ventral tegmental area. NeuroImage 237:118136. doi: 10.1016/j.neuroimage.2021.118136 33951514

[ref47] Sun J. (2023). Problematic insta user: motivations and the mediating role of fear of missing out among young adults. Curr. Psychol. 42, 14919–14928. doi: 10.1007/s12144-022-02775-y

[ref48] Sun Y. Zhang Y. (2021). A review of theories and models applied in studies of social media addiction and implications for future research. Addict. Behav. 114:106699. doi: 10.1016/j.addbeh.2020.106699 33268185

[ref49] Tian X. Bi X. Chen H. (2023). How short-form video features influence addiction behavior? Empirical research from the opponent process theory perspective. Inf. Technol. People 36, 387–408. doi: 10.1108/ITP-04-2020-0186

[ref50] Wang K. Scherr S. (2022). Dance the night away: how automatic TikTok use creates pre-sleep cognitive arousal and daytime fatigue. Mob. Media Commun. 10, 316–336. doi: 10.1177/20501579211056116

[ref51] Yan T. Su C. Xue W. Hu Y. Zhou H. (2024). Mobile phone short video use negatively impacts attention functions: an EEG study. Front. Hum. Neurosci. 18:1383913. doi: 10.3389/fnhum.2024.1383913, 38993329 PMC11236742

[ref52] Yang Z. (2023). Why adolescents are addicted to social media. J. Educ. Humanit. Soc. Sci. 8, 1430–1436. doi: 10.54097/ehss.v8i.4498

[ref53] Ye J.-H. Wu Y.-F. Nong W. Wu Y.-T. Ye J.-N. Sun Y. (2023). The association of short-video problematic use, learning engagement, and perceived learning ineffectiveness among Chinese vocational students. Healthcare 11:161. doi: 10.3390/healthcare11020161, 36673529 PMC9858663

[ref54] Ye J. H. Wu Y. T. Wu Y. F. Chen M. Y. Ye J. N. (2022). Effects of short video addiction on the motivation and well-being of Chinese vocational college students. Front. Public Health 10:847672. doi: 10.3389/fpubh.2022.847672, 35619803 PMC9127725

[ref55] Ye J. H. Zheng J. Nong W. Yang X. (2025). Potential effect of short video usage intensity on short video addiction, perceived mood enhancement ('TikTok brain'), and attention control among Chinese adolescents. Int. J. Ment. Health Promot. 27, 271–286. doi: 10.32604/ijmhp.2025.059929

[ref56] Yu X. Wang Y. Liu F. (2022). Language learning motivation and burnout among English as a foreign language undergraduate: the moderating role of maladaptive emotion regulation strategies. Front. Psychol. 13:808118. doi: 10.3389/fpsyg.2022.808118, 35185728 PMC8854853

[ref57] Zhang N. Hazarika B. Chen K. Shi Y. (2023). A cross-national study on the excessive use of short-video applications among college students. Comput. Hum. Behav. 145:107752. doi: 10.1016/j.chb.2023.107752

