# Supplementary material for: From short-form video addiction to burnout: exploring the role of emotional enhancement (TikTok brain) and perceived video-attributed attentional decline through the I-PACE model
Source: Front Psychol. 2026 Aug 20;17:1910124. doi: 10.3389/fpsyg.2026.1910124 (PMC13538871; doi:10.3389/fpsyg.2026.1910124)
Supplement: Supplementary file 1 [file Table_1.DOCX]

**Appendix A**

**TABLE A** Measurements

| **No.** | **Short-form video addiction** |
| --- | --- |
| 1 | I watch short-form videos longer than I originally intended. |
| 2 | I neglect tasks or responsibilities to spend time watching short videos. |
| 3 | The excitement or anticipation of watching short videos outweighs my interest in other social interactions. |
| 4 | I have been complained about or criticized by others due to my short video usage. |
| ~~5~~ | I have been late, left early, or missed school because of watching short videos. |
| ~~6~~ | My academic performance has declined due to watching short videos. |
| 7 | I get upset when someone interrupts me while I’m watching short videos. |
| 8 | I sacrifice my sleep at night to watch short videos. |
| 9 | Even after closing the platform, I can’t stop thinking about the short video content. |
| 10 | I feel down when I don’t watch short videos. |

| **No.** | **Emotional enhancement (TikTok Brian)** |
| --- | --- |
| 11 | I often need to watch TikTok short videos to make me feel happier. |
| 12 | I often need to watch TikTok short videos to feel more energized. |
| 13 | I often need to watch TikTok short videos to enhance my mood. |
| 14 | I often need to watch TikTok short videos to experience quick satisfaction. |
| 15 | I often need to watch TikTok short videos to immerse myself in them. |
| 16 | I often need to watch TikTok short videos to feel joy and excitement. |
| 17 | I often need to watch TikTok short videos to forget my troubles. |
| 18 | I often need to watch TikTok short videos to gain a sense of happiness. |
| 19 | to feel a heightened emotional state. |

| **No.** | **Perceived video-attributed attentional decline** |
| --- | --- |
| 20 | Since using short video apps, I find it hard to focus on one activity for a long time. |
| 21 | Since using short video apps, it often takes me some time to refocus my attention. |
| 22 | Since using short video apps, I find it difficult to calm myself down. |
| 23 | Since using short video apps, I struggle to concentrate on my goals. |
| 24 | Since using short video apps, I often get distracted from what I am doing. |
| 25 | Since using short video apps, I find it hard to regain focus after being interrupted. |

| **No.** | **English-Learning burnout** |
| --- | --- |
| 26 | I feel emotionally drained by my English studies. |
| 27 | I feel tired when I get up in the morning and I have to face English study another day at school. |
| 28 | Studying or attending an English class is really a strain for me. |
| 29 | I feel burned out from my English studies. |
| 30 | I have become less enthusiastic about my English studies. |
| 31 | I have become increasingly indifferent to my English studies. |
| 32 | I’ve become more cynical about the potential usefulness of my English study. |
| 33 | I believe that I don’t make an effective contribution to the English classes that I attend. |
| 34 | I don’t feel stimulated when I reach my goals in English study. |
| 35 | During English class I don’t feel confident that I am effective in getting things done. |

**TABLE B** Inner VIF values

| **Constructs** | **ELB** | **TB** | **PAD** | **SFVA** |
| --- | --- | --- | --- | --- |
| **ELB** |  |  |  |  |
| **TB** | 1.068 |  |  |  |
| **PAD** | 1.129 |  |  |  |
| **SFVA** | 1.120 | 1.000 | 1.000 |  |

**TABLE C** Outer VIF values

| **Items** | **VIF** |
| --- | --- |
| PAD1 | 2.080 |
| PAD2 | 2.015 |
| PAD3 | 2.013 |
| PAD4 | 2.220 |
| PAD5 | 1.857 |
| PAD6 | 2.288 |
| ELB1 | 2.667 |
| ELB10 | 2.240 |
| ELB2 | 2.654 |
| ELB3 | 2.457 |
| ELB4 | 2.521 |
| ELB5 | 2.595 |
| ELB6 | 2.368 |
| ELB7 | 1.821 |
| ELB8 | 2.003 |
| ELB9 | 2.073 |
| SFVA1 | 1.876 |
| SFVA10 | 1.327 |
| SFVA2 | 2.308 |
| SFVA3 | 2.207 |
| SFVA4 | 2.003 |
| SFVA5 | 2.879 |
| SFVA6 | 2.340 |
| SFVA7 | 1.993 |
| SFVA8 | 1.147 |
| SFVA9 | 1.207 |
| TB1 | 2.511 |
| TB2 | 2.475 |
| TB3 | 2.479 |
| TB4 | 2.167 |
| TB5 | 2.444 |
| TB6 | 2.431 |
| TB7 | 2.510 |
| TB8 | 2.560 |
| TB9 | 2.373 |

**TABLE D**

| **Model** | **SFVA→ELB**  **β** | **t-Value** | **p** | **ELB**  **R^2^** |
| --- | --- | --- | --- | --- |
| **Original model** | 0.301*** | 6.133 | <0.001 | 0.175 |
| **SFVA items 5–6 removed** | 0.297*** | 6.071 | <0.001 | 0.172 |
